# Identification and Validation of the Immune Subtypes of Lung Adenocarcinoma: Implications for Immunotherapy

**DOI:** 10.3389/fcell.2020.00550

**Published:** 2020-07-03

**Authors:** Yang Song, Shi Yan, Weina Fan, Mengyan Zhang, Wei Liu, Hailing Lu, Mengru Cao, Chenguang Hao, Lin Chen, Fanglin Tian, Yuning Zhan, Li Cai, Ying Xing

**Affiliations:** ^1^Department of Orthopedic Surgery, The Second Affiliated Hospital of Harbin Medical University, Harbin, China; ^2^Department of Medical Oncology, Harbin Medical University Cancer Hospital, Harbin, China; ^3^School of Life Sciences and Technology, Computational Biology Research Center, Harbin Institute of Technology, Harbin, China

**Keywords:** lung adenocarcinoma, tumor immune microenvironment, immune subtypes, clinicopathological features, molecular and cellular characteristics

## Abstract

Lung adenocarcinoma (LUAD) is a devastating disease with poor patient survival. Cancer immunotherapy has revolutionized the treatment of LUAD, but only a limited number of patients effectively respond to this treatment. Thus, the work to elucidate the LUAD immune heterogeneity could be crucial in developing new immunotherapeutic strategies with better efficacy. Non-negative matrix factorization-based deconvolution was performed to identify robust clusters of 489 LUAD patients in The Cancer Genome Atlas (TCGA) and verify their reproducibility and stability in an independent LUAD cohort of 439 patients from the Gene Expression Omnibus (GEO). We used the graph learning-based dimensionality reduction to visualize the distribution of individual patients. In this study, four reproducible immune subtypes, Clusters 1–4 (C1–C4) associated with distinct gene module signatures, clinicopathological features, molecular and cellular characteristics were identified and validated. The immune-cold subtype, C3, was associated with the Dead event, the most advanced T stage, N stage, TNM stage and the worst prognosis for LUAD patients. Moreover, C3 exhibited the lowest infiltrating levels of B cells, T cell receptor (TCR) repertoire diversity and the highest level of neoantigen and mutation rate among C1–C4. On the other hand, the immune-hot subtype (C4) exhibited the highest infiltration of six types of infiltrating immune cells as well as the greatest leukocyte fraction, TCR and B cell receptor (BCR) repertoire diversity. C1 and C2 subtypes showed diverse clinicopathological and immunological features. Finally, our investigations discovered a complex immune landscape with a scattered immune subtype profile. This work may help inform immunotherapeutic decision-making and design advanced immunotherapy strategies for the treatment of lung cancer.

## Introduction

Lung cancer is a devastating disease worldwide because it has the highest morbidity and mortality rate among all cancers (Bray et al., 2018). Non-small cell lung cancer (NSCLC) accounts for approximately 85% of lung cancers, and lung adenocarcinoma (LUAD) is the most common histological type of NSCLC (Herbst et al., 2008; Sivakumar et al., 2017). Although many therapeutic strategies including surgery have shown immense progress, the 5-year survival rate of LUAD is very low (Heist and Engelman, 2012). Immunotherapy, such as anti-PD-1 therapy, has been proven to have enormous potential in the treatment of LUAD; however, immune agents benefit only a subset of patients (Alatrash et al., 2013; Garon et al., 2015). Thus, it is urgent to identify novel immune subgroups correlated with treatment response (Binnewies et al., 2018).

An increasing number of studies have found that the immune-related features of cancers such as the intensity of CD8+ T cell infiltrates, leukocyte fraction, T cell receptor (TCR) and B cell receptor (BCR) repertoire (Tang et al., 2016; Li et al., 2019; Zeng et al., 2019) were correlated with immunotherapeutic responsiveness in various cancers, including lung cancer, however, the immune-related features themselves alone are not a sufficient predictor of response to immunotherapeutic intervention (Riaz et al., 2017; Moya-Horno et al., 2018). Multiple changes in the tumor immune microenvironment (TIME) were able to powerfully impact and even determine the heterogeneous response to immunotherapy (Binnewies et al., 2018).

Encouragingly, genomic and transcriptomic data based on The Cancer Genome Atlas (TCGA) have been employed to study the TIME, investigation of the immune landscape and definition of immune subtypes of human cancer comprising 33 diverse cancer types (Binnewies et al., 2018; Thorsson et al., 2018). Previous studies reported that the distribution of immune subtypes was tissue-specific within the different tumor types (Thorsson et al., 2018; Li et al., 2019). In addition, various immune subtypes and landscapes in TCGA set were illuminated in lung squamous cell carcinoma, head and neck squamous cell carcinoma, cervical squamous cell carcinoma, esophageal squamous cell carcinoma, papillary thyroid cancer, gastric cancer, breast cancer (He et al., 2018), and other cancers (Park et al., 2017; Kim et al., 2018; Canning et al., 2019; Chen et al., 2019; Li et al., 2019; Lin et al., 2019). The three LUAD subtypes including the terminal respiratory unit, proximal proliferative, and proximal inflammatory subtypes, displayed differences in the tumor immune landscape (Wilkerson et al., 2012; Faruki et al., 2017). Nevertheless, to our knowledge, the LUAD tumor landscape and immune subtypes impacting clinical outcomes remain largely unknown.

In this study, the proposed computational algorithms were applied to discover (Kim et al., 2018; Chen et al., 2019; Zeng et al., 2019), and validate four robust immune clusters in LUAD based on immune-related genes (IRGs) (Li et al., 2019). Next, we characterized the four immune subtypes. As a result, each immune subtype was correlated with distinct gene module signatures, clinicopathological signatures, molecular and cellular features. Ultimately, an immune landscape composed of both continuous spectrum and discrete clusters across LUAD patients was delineated.

## Materials and Methods

### Discovery and Validation of the Immune Subtypes

This study was approved by the Institutional Ethics Committee of Harbin Medical University, China. The discovery cohort consisted of 489 patients with LUAD from TCGA (Supplementary Table S1). An independent meta-cohort from Gene Expression Omnibus (GEO) (GSE68465) was used for further validation (Supplementary Table S2). Based on IRGs, we identified robust immune clusters of patients and immune-related signatures by non-negative matrix factorization (NMF) clustering analysis (Supplementary Methods; Kim et al., 2018; Chen et al., 2019).

### Evaluating Clinicopathological, Molecular and Cellular Features Correlated With the Immune Subtypes

First, we assessed the proportion of immune subtypes and immune related signatures in LUAD patients from TCGA. Relationships between clinicopathological features and immune subtypes were analyzed by parametric (Chi-square test) and non-parametric (Fisher’s exact) assessments where appropriate. Overall survival (OS) and progression-free survival (PFS) rate were analyzed according to the Kaplan–Meier method, and differences between survival distributions were assessed with the log-rank test. The prognostic effect of immune-related signatures was determined by Cox regression. Receiver operating characteristic curves (ROCs) were drawn for the predicted 1, 3, 5-year OS based on the nearest neighbor method, and area under the curve (AUC) was calculated. ANOVA was used to detect the association between immune subtypes and all kinds of immune-related molecular and cellular features (Thorsson et al., 2018; Supplementary Methods).

### Immune Landscape Analysis

Using the reduceDimension function of the Monocle package with a Gaussian distribution, graph learning-based dimensionality reduction analysis was performed as previously described (Trapnell et al., 2014; Li et al., 2019). The discriminative dimensionality reduction with trees (DDRTree) was used to conduct dimension reduction (Qiu et al., 2017). In summary, we projected data points in a high-dimensional space to latent points in the low-dimensional space in the form of a tree structure (Qi et al., 2017; Wang and Mao, 2019). The presented immune landscape establishes a linkage among patients in a nonlinear manifold that might make up for the discrete immune subtypes in the linear Euclidean space (Supplementary Methods).

## Results

### Immune Subtypes and Gene Module Signatures in LUAD

The previous study by Li et al. (2019) integrated single-cell and bulk tumor RNA-seq data and presented a recognizable datasheet of 1989 IRGs in squamous cell carcinoma. The corresponding mRNA expression of 1318 genes of these IRGs was intermediate or high in LUAD based on TCGA and GEO (GSE37745 and GSE3141) data sets. Furthermore, the 376 genes impacting the prognosis of LUAD patients by univariate Cox regression analysis were included in subsequent analysis (Supplementary Table S3).

**FIGURE 1:**
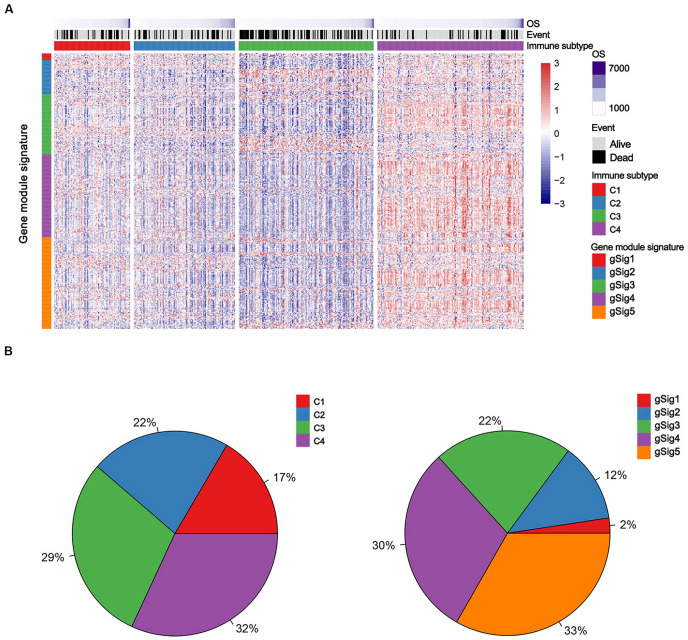
The immune subtypes and gene module signatures in the TCGA LUAD cohort. **(A)** Columns and rows represent patients and genes, respectively. Patients (TCGA dataset, *n* = 489) are arranged based on their immune subtypes and genes are ordered based on the gene module signatures. OS and survival events are annotated for each patient. **(B)** The distribution of immune subtypes and gene module signatures in the TCGA cohort. C1, Cluster 1; C2, Cluster 2; C3, Cluster 3; C4, Cluster 4.

Non-negative matrix factorization clustering results showed that four robust clusters (C1–C4) were identified in the TCGA discovery cohort (Figures 1A,B and Supplementary Figure 2). Simultaneously 5 was regarded as the optimal gene module number according to the Bayesian Information Criterion index, providing gene module signatures 1–5 (defined as gSig1–5, Figures 1A,B, Supplementary Figure 3, and Supplementary Table S4).

### The Correlation Between Immune Subtypes and Gene Module Signatures

**FIGURE 2:**
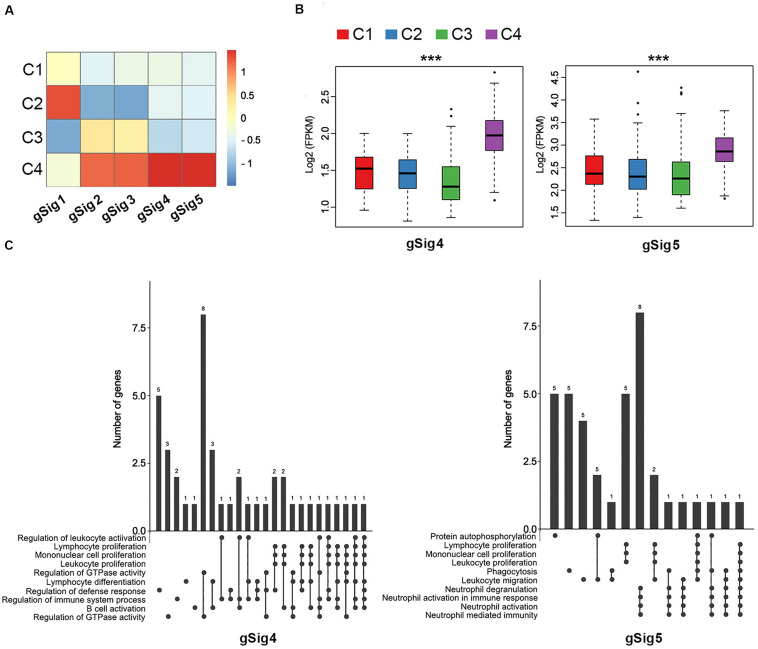
The correlation between immune subtypes and gene module signatures in TCGA. **(A)** The heatmap of expression of the gene module signatures among the C1–C4 immune subtypes. **(B)** The expression of gSig4 and gSig5 in the C1–C4. **(C)** UpSet plot shows the significant enrichment of Gene Ontologies (GO) of gSig4 and gSig5. The bar chart above represents the number of genes contained in each type of group. The dotted line at the bottom right shows the types of events contained in the group. ***P < 0.001.

Every immune cluster was correlated with a specific gene module signature (Figure 1A). The linear correlation coefficients between immune subtypes and gene module signatures are shown in Figure 2A. The immune subtypes correlated with gene module signatures (Figure 2B, Supplementary Figure 4, and Supplementary Table S5). GO enrichment analysis indicated that gSig4 and gSig5 were positively associated with the mediation of immune activation (Figure 2C and Supplementary Table S6). On the other hand, gSig1, gSig2, and gSig3 were closely related to metabolism, cell architecture and signal transduction (Supplementary Figure 5 and Supplementary Table S6). Additionally, our gene modules of gSig4 and gSig5 mostly mapped the previously proposed gene module “inflammation,” which was regarded as a subtype with superior prognosis relative to other subtypes by Li et al. (2019), suggesting that gSig4 and gSig5 were associated with inflammation and improved survival (Supplementary Figure 6 and Supplementary Table S7). As expected, with the increases of clinical T stage, N stage, M stage, and TNM stage the expression levels of both gSig4 and gSig5 were significantly elevated in LUAD patients (Supplementary Figures 7A–D). We also found that the high expression levels of gSig4 and gSig5 were associated with female gender and, importantly, favorable survival of LUAD patients (Supplementary Figures 7E,F). The expression level of gSig1–3 was not associated with any clinicopathological or prognostic characteristic (Supplementary Figure 8).

Of note, C3 had the lowest expression in the gene modules of gSig4 and gSig5, suggesting an immune-cold phenotype, while C4 had the highest expression in the gene modules of gSig4 and gSig5, suggesting an immune-hot phenotype (Figure 2B). In addition, we found that C3 also had the lowest expression in the gene modules of gSig1, and C4 had the highest expression in the gene modules of gSig2 and gSig3 (Supplementary Figure 4).

### The Clinicopathological Signature of the Immune Subtypes

**FIGURE 3:**
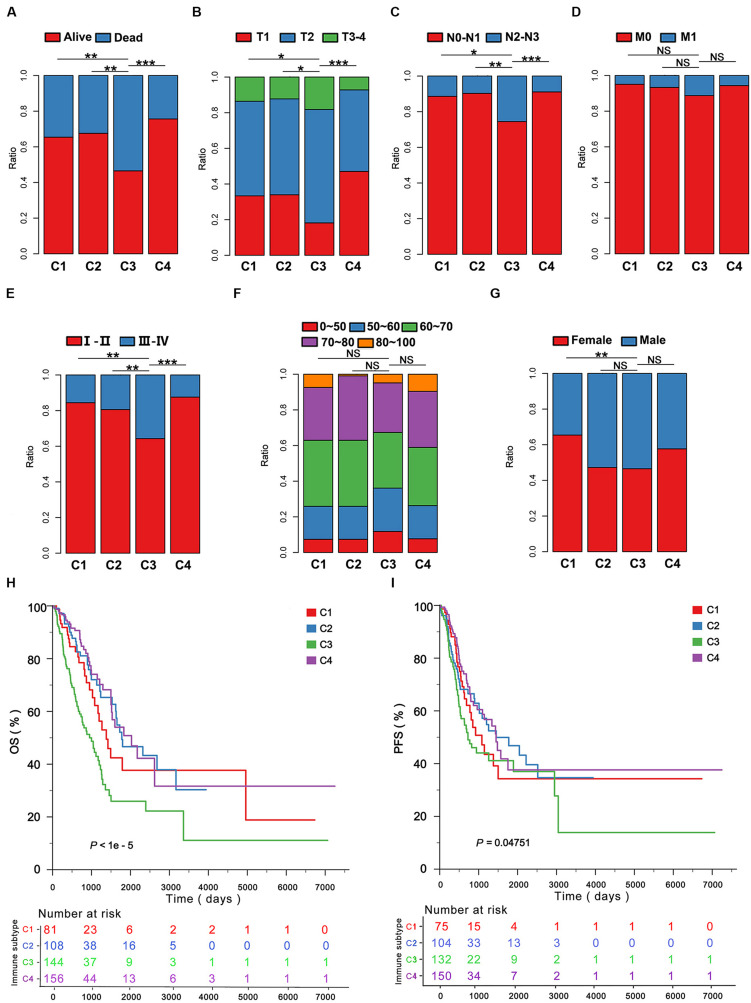
The clinicopathological signatures of the immune subtypes in TCGA. The patients were classified according to the clinical features including **(A)** survival event, **(B)** T stage, **(C)** N stage, **(D)** M stage, **(E)** TNM stage, **(F)** age, **(G)** gender in the immune subtypes. **(H,I)**, Five-year Kaplan–Meier curves for overall survival (OS) and progression-free survival (PFS) of LUAD patients from the TCGA cohort stratified by the immune subtypes. The *P*-value was calculated by the log-rank test among subtypes. *P < 0.05, **P < 0.01, ***P < 0.001.

Among all subtypes C3 was associated with the Dead event (Figure 3A), the most advanced T stage (Figure 3B), N stage (Figure 3C), TNM stage (Figure 3E) compared to C1, C2, and C4 in the LUAD cohort of TCGA. C3 was not well associated with M stage, age and gender compared with the other immune subtypes (Figures 3D,F,G). Furthermore, the OS and PFS yielded the worst prognosis for the LUAD patients with C3 compared with those with C1, C2, and C4 (Figures 3H,I). Moreover, we found C3 could effectively predict 1, 3, and 5 years OS by ROCs (*P* < 0.05; Supplementary Figure 9A). To validate our findings in the TCGA cohort, we investigated the reproducibility of the immune subtypes in an independent GEO cohort (GSE68465). Using the in-group proportion (IGP) and Pearson correlation among centroids of gene module scores, the consistency was found in subtype identification at both patient and subtype levels in the discovery and validation cohorts (*P* < 1e-5). In line with the finding from TCGA, C1–C4 were identified, and C3 predicted the worst survival among the immune subtypes (Supplementary Figures 9B,C).

In this study, we found that the immune subtype C3 was a robust prognostic biomarker.

### Cellular and Molecular Features of LUAD Immune Subtypes

**FIGURE 4:**
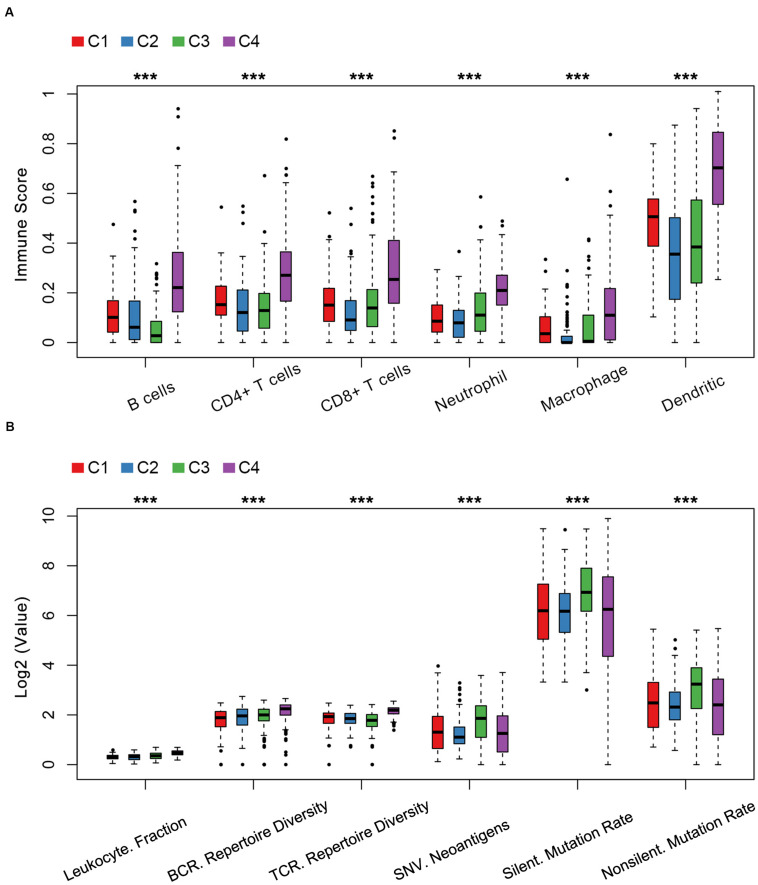
Cellular and molecular features of LUAD immune subtypes in TCGA. **(A)** Immune score distribution of six immune cells in immune subtypes among the C1–C4 immune subtypes. **(B)** The index distribution of leukocyte fraction, BCR/TCR repertoire diversity, single nucleotide variant (SNV) neoantigen, silent mutation rate and non-silent mutation rate among the C1–C4 immune subtypes. Kruskal–Wallis test was used. ***P < 1e-5.

The relationship between cellular features and immune subtypes was revealed. C4 was enriched with immune cells including activated B cells, CD4+ T cells, CD8+ T cells, neutrophil cells, macrophages and dendritic cells by the tumor immune estimation resource (TIMER) (Figure 4A and Supplementary Table S8). In contrary, the C3 subtype exhibited the least number of B cells, in line with the unfavorable prognostic significance of C3.

Previous studies reported that the analysis of leukocyte fraction, TCR and BCR repertoire diversity inference are several important techniques to access the immune landscape (Thorsson et al., 2018; Li et al., 2019). Next, we explored the relationship between the immune subtypes and molecular features. The immune subtype C3 was associated with a lower TCR repertoire diversity, a higher neoantigen load and a higher rate of silent mutation and non-silent mutation compared to C1, C2, and C4 (Figure 4B and Supplementary Table S9). Consistent with an immune-hot phenotype, tumors in C4 had the highest leukocyte fraction, TCR repertoire diversity and BCR repertoire diversity compared to C1, C2, and C3 (Figure 4B and Supplementary Table S9).

### Immune Landscape of LUAD

**FIGURE 5:**
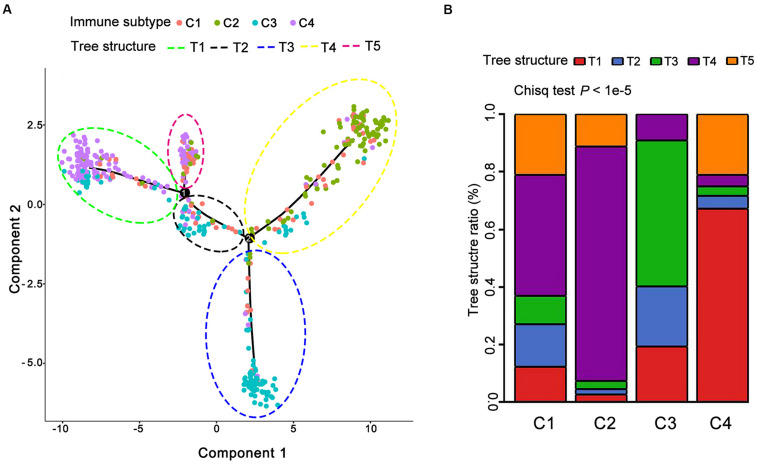
The immune landscape of LUAD in TCGA. **(A)** Each point represents a patient with colors corresponding to the immune subtype defined previously. **(B)** Distribution of five tree structures (defined as T1–T5) among C1–C4.

Next, we sought to make visualization of the immune landscape with the function plot cell trajectory with the color corresponding to the immune subtype identified above. In detail, we employed a graph-based learning approach to perform dimension reduction based on previously described procedures (Qi et al., 2017; Wang and Mao, 2019). The results demonstrated that 489 individual LUAD patients were cast into a manifold with sparse tree structures and depicted the immune landscape of LUAD based on the TCGA database (Figure 5A). The location of individual patients in the five tree structures signified the comprehensive characterization of TIME in the distinct immune subtypes. In lines with the identified immune subtypes above, we found that many patients were divided into distinct clusters and there was a significant overlap of patients between five tree structures and four identified immune subtypes (*P* < 1e-5, Figure 5B and Supplementary Table S10). For example, C4 mainly gathered on the end of horizontal coordinate on left, while most of C3 was located on the end of the vertical axis at the bottom (Figure 5A). Consequently, these findings regarding the distribution of LUAD immune subtypes suggested the reproducibility of our defined immune subtypes.

## Discussion

Immunotherapy has shown a considerable clinical success in the treatment response of many LUAD patients; however, when provided with the same immunotherapeutic intervention, little or no clinical benefit is unsatisfactorily found in the many more patients (Forde et al., 2018; Passiglia et al., 2018; Almutairi et al., 2019). As technology has advanced in techniques such as high-resolution single-cell RNA sequencing, the diversity and complexity of the immune context of TIME impacted tumor initiation and immunotherapeutic responsiveness in lung cancer (Binnewies et al., 2018; Clarke et al., 2019). In this study, four reproducible immune subtypes of LUAD were identified, independently validated and comprehensively characterized. We discovered that each of the immune subtypes was associated with distinct gene module signatures, clinicopathological features, and accordingly demonstrated widely different patterns in tumor genetic aberrations, molecular and cellular characteristics. The parsing of four distinct classes of TIME in LUAD is likely to help to benefit the identification of patient populations responsive to current immunotherapy and immune therapeutic modulation (Binnewies et al., 2018).

This study was different from recent immune subtype reports on squamous carcinomas and pan-cancer (Thorsson et al., 2018; Li et al., 2019), and we only focused on LUAD, which displays common etiology and histological characterization. A previous study by Li et al. (2019) identified six immune subtypes across four major cancer types, namely, head and neck, lung, cervical and esophageal squamous cell carcinoma, and showed that approximately 75% of lung squamous cell carcinomas were clustered into subtypes 1 and 5, which predict almost identical clinical outcomes, while a majority (∼80%) of cervical squamous cell carcinomas were clustered into subtypes 4 and 6 (Li et al., 2019). The results reported by Thorsson et al. (2018) also revealed that the distribution of immune subtypes across 33 kinds of cancer tissues was tissue-specific. In addition, there is also evidence that immune prognostic value varied according to histology (Chifman et al., 2016). There is a growing need to explore distinct subclasses of TIME immune subtypes in individual tumor types, which correlated with the likelihood of response to immunotherapeutic intervention targeting a specific type of cancer.

A great deal of studies have demonstrated the significance of IRGs in recognition, surveillance, clinical prognosis and chemotherapeutic and immunotherapeutic responsiveness of human cancer (Gnjatic et al., 2017; Li B. et al., 2017; Park et al., 2017; Prat et al., 2017; Anichini, 2019; Lin et al., 2019). In lung cancer, based on IRG pairs, the proposed clinical-immune signature as a potential biomarker was able to predict OS for patients with nonsquamous NSCLC (Li B. et al., 2017). IRGs or signatures related to the response and PFS after immunotherapy are found in several types of cancer including lung cancer (Prat et al., 2017). The IRGs in our work were derived from a previous study by Li et al. (2019) in which they focused on five different categories: (1) immune cell-specific genes derived from single-cell RNA-seq data, (2) genes of co-stimulatory and co-inhibitory molecules, (3) genes of cytokine and cytokine receptors, (4) genes involved in antigen processing and presentation, and (5) other IRGs. Compared with the IRGs in previous studies in IRGs, a larger number of and more diverse IRG datasets were included in the study.

In line with the finding by Li et al. (2019), the immune-cold subtype that we defined had the lowest expression in the gene modules of gSig4 and gSig5, which mostly mapped the previously proposed gene module “inflammation,” while C4 had the highest expression in the gene modules of gSig4 and gSig5, suggesting an immune-hot phenotype. Our current study showed that the immune-cold subtype related to Dead event, the most advanced T stage, N stage, and TNM stage. In line with our studies, the Exhausted Immune Class was associated with late pathologic T-status in head and neck squamous cell carcinoma (Chen et al., 2019), and the low ImmuneScore group was significantly associated with advanced T stage, lymph node metastasis, and advanced AJCC stage in papillary thyroid cancer (Kim et al., 2018; Na and Choi, 2018; Lin et al., 2019), There was a significant association between our immune subtypes and clinicopathological signatures such as pathological stage, suggesting that the immune subtypes might influence on tumor initiation and progression.

Here, we found that the immune-cold subtype was reproducibly associated with the worst prognosis for LUAD patients. In agreement with our conclusion, the previously described role of the immune-cold subtype or the subclass exhausted immune responses as an indicator of poor survival (Park et al., 2017; Kim et al., 2018; Na and Choi, 2018; Chen et al., 2019; Li et al., 2019; Lin et al., 2019). Although there was a trend in differences in survival rate between the immune-hot subtype and other subtypes, there was no significant prognostic value for C4. This result might be explained by the limitation of our study in that some IRGs that impacted TIME of LUAD were not included in our study because of the gene expression profiles from the squamous cell carcinoma data sets (Li et al., 2019). Future studies will be performed using a combination of gene expression profiles from multiple data sets and used larger number of IRGs for LUAD.

Our results further demonstrated that the immune-cold subtype exhibited the lowest infiltrating levels of B and CD4+ T cells, while the immune-hot subtype disclosed the highest infiltration of six types of infiltrating immune cells among four immune clusters using the Tumor Immune Estimation Resource (TIMER). Instead of CIBERSORT (Gentles et al., 2015; Li et al., 2019), PRECOG (Gentles et al., 2015) and TCIA (Charoentong et al., 2017) utilized in the previous studies, TIMER (Li T. et al., 2017), which is a comprehensive and an innovative and computational method that integrates and deconvolves multi-dimensional datasets, was used in our study. It is well known that immune infiltrates might influence clinical responsiveness and be heterogeneous in different patients with LUAD (Li T. et al., 2017; Liu et al., 2017). For instance, tumors lacking in B cells predicted unfavorable outcomes for LUAD patients at an early clinical stage (Liu et al., 2017).

Our current results indicated that the immune subtypes have the potential to act as predictors of immune cell infiltration elevation. Moreover, the immune-cold subtype was linked to the lowest level of TCR repertoire diversity, while the immune-hot subtype was correlated with the greatest leukocyte fraction and TCR/BCR repertoire diversity, which was consistent with previous reports (Li et al., 2019). The positive correlation of leukocyte fraction, TCR/BCR repertoire diversity and upregulation of the checkpoint inhibitors on tumors and immune cells was observed in previous studies (Riaz et al., 2017).

It is imaginable that the patients with the immune-hot subtype of LUAD would be more likely to respond to immunotherapy, while the patients with the immune-cold subtype of LUAD would be less likely benefit from immunotherapy than patients with other LUAD subtypes. Our results should be noted that our findings require further validation in immunotherapy-treated LUAD tumors. The findings should be interpreted with this limitation in mind.

Recent publication highlighted the potential limitations of studies using TCGA database without considering the effect of tumor heterogeneity (Li et al., 2014; Jia et al., 2018). Sequencing more tumors with the TCGA approach of single time-point sampling can neither capture the heterogeneity between different parts of the same tumor nor catch the heterogeneity (Li et al., 2014). There can be no doubt that intratumoral spatial and temporal heterogeneity becomes a confounding factor to this study. Different methods to enhance identifying cancer targets may be necessary, such as single cell technology (Hu et al., 2020), real time imaging of cancer cells with a biological global positioning system (Li et al., 2010), and cross-referencing big data sets (Li et al., 2014). These methods are offered as ways to address sampling discrepancies in the face of tumor heterogeneity.

Taken together, our findings identified four immune subtypes of LUAD that relate to distinct clinicopathological, cellular and molecular characteristics. Immune subtyping could be utilized to identify LUAD patients who will be affected by TIME and might guide a personalized approach to cancer immunotherapy.

## Data Availability Statement

All datasets generated for this study are included in the article/Supplementary Material.

## Author Contributions

YS, SY, and WF designed the study, interpreted the data, analyzed the results, and were major contributors in writing and revising the manuscript. MZ provided the technical support. WL, HL, MC, CH, LCh, FT, and YZ helped with manuscript writing, review, and revision. LCa and YX assisted with manuscript review and revision. All authors read and approved the final manuscript.

## Conflict of Interest

The authors declare that the research was conducted in the absence of any commercial or financial relationships that could be construed as a potential conflict of interest.
