# Supplementary material for: Identification and Validation of the Immune Subtypes of Lung Adenocarcinoma: Implications for Immunotherapy
Source: Front Cell Dev Biol. 2020 Jul 3;8:550. doi: 10.3389/fcell.2020.00550 (PMC7348081; doi:10.3389/fcell.2020.00550)
Supplement: Supplementary file 2 [file Data_Sheet_1.docx]

Supplementary Material

**Supplement methods**

**Study design**

The construction scheme of immune subtypes and gene module signatures was systematically evaluated in Supplementary Figure S1. Initially, in terms of prior knowledge about the immune genes ([Li et al., 2019](#_ENREF_11)), to characterize intratumoural immune states, we identified immune- and prognosis-related gene sets based on LUAD in the Cancer Genome Atlas (TCGA) and the Gene Expression Omnibus (GEO) and then used cluster analysis to identify immune subtypes and gene module signatures of immune-related gene (IRGs) sets. The robust immune subtypes were also validated in the GEO (GSE68465) dataset. Next, we explored the clinical and immunological features of immune subtypes and gene signatures. To this end, we established the immune landscape in view of graph structure learning based dimensionality reduction analysis.

**Patient selection and data preprocessing**

For the TCGA discovery cohort, a total of 522 LUAD RNA-seq data samples were systematically searched from the Pan-Cancer Atlas project. The latest clinical follow-up information and some molecular and cellular immunological characteristics were also retrieved for the patients in the discovery cohort (Supplementary Table S1). These characteristics comprised tumor genetic mutation, neoantigens, main immune cell composition, TCR/BCR Shannon, and fraction of leukocyte ([Thorsson et al., 2018](#_ENREF_22)), The operation of TCGA data preprocessing included three steps: 1) remove samples without clinical data and overall survival (OS) < 30 days; 2) remove normal tissue sample data; and 3) remove IRGs with the fragments per kilobase of gene per million fragments mapped FPKM < 1 from half of the samples.

For the Gene Expression Omnibus (GEO), the GSE37745 dataset and GSE3141 dataset were used to screen the IRGs in LUAD. In addition, the GSE68465 dataset (Supplementary Table S2) was applied as a validation cohort. The preprocessing steps for GSE37745, GSE3141 and GSE68465 data: 1) removed normal tissue sample data; 2) converted OS data from years or months to days; 3) removal of the samples with OS < 30 days; and 4) mapping the chip probe to the human gene SYMBOL by using the Bioconductor package. The two Affymetrix (U1332 plus) chip datasets were both normalized with RMA algorithm ([Gautier et al., 2004](#_ENREF_6)); 5) remove IRGs with the fragments per kilobase of gene per million fragments mapped FPKM < 1 from half of the samples.

**Collection of immune-related genes**

First, after data processing, the genes with no expression and low expression in LUAD were excluded, and 1318 IRGs were screened for study. Last, the 376 genes impacting prognosis of LUAD patients by univariate Cox regression analysis were included in subsequent analysis (Supplementary Table S3) (Log-rank *P* < 0.05).

**Determination of immune subtypes and gene signatures**

To identify robust immune subtypes, the nonnegative matrix factorization (NMF) algorithm groups the samples into clusters, for each factorization rank from 2 to 10 ([Brunet et al., 2004](#_ENREF_2)). To obtain the optimal clusters, we evaluated the stability of factorization by the cophenetic correlation coefficient ([Brunet et al., 2004](#_ENREF_2)), residual sum of squares (RSS) ([Hutchins et al., 2008](#_ENREF_9); [Fogel et al., 2016](#_ENREF_4)) and silhouette statistic ([Lovmar et al., 2005](#_ENREF_13)) of *k*. An optimal *k* (*k* = 2-10) should correspond to a low RSS, a high cophenetic correlation coefficient and high silhouette statistic. Moreover, a clustering algorithm ([Marisa et al., 2013](#_ENREF_14)) which was based on the Euclidean distance metric, was also used to determine the number of clusters in the TCGA cohort ([Seiler et al., 2010](#_ENREF_19)). We performed the Bayesian Information Criterion (BIC) for clustering 376 gene sets and the number of clusters corresponding to the lowest value of BIC was the best classification ([Fontes et al., 2013](#_ENREF_5)). In this study, we Gene Ontology (GO) terms by the DAVID Bioinformatics Resources (v6.8) ([Hochberg et al., 1990](#_ENREF_7); [Huang da et al., 2009](#_ENREF_8); [Zeng et al., 2019](#_ENREF_24)). The average expression level of all genes in a specific module represented the score for gene signature.

**Validation of the immune subtypes in the GEO dataset**

In brief, the in-group-proportion (IGP) statistic was used to quantitatively assess the similarity and reproducibility of the proposed immune subtypes between discovery and validation cohorts, at individual patient level as well as for gene expression patterns. A high IGP for a subtype corresponds to a reproducible partition of patients for that subtype. To estimate IGP, we calculated the centroid of each immune subtype of the shared immune-related genes in the TCGA discovery cohort. Then, each sample in the GEO validation cohort was assigned to an immune subtype whose centroid had the highest. Pearson correlation with the sample, and the IGP was estimated for each subtype in the validation cohort. In other words, the IGP measures the proportion of patients classified to an immune subtype whose nearest neighbors were also classified to the same immune subtype. The statistical significance of IGP was estimated with 500 permutations (package clusterRepro) ([Kapp et al., 2007](#_ENREF_10)).

**Immune-related features**

The main immune cell compositions were assessed, which consisted of B cells, CD4+ T cells, CD8+ T cells, neutrophils, macrophages and DCs. To quantify the proportions of the 6 immune cells in the LUAD samples, we used the Tumor Immune Estimation Resource (TIMER) ([Li et al., 2017](#_ENREF_12)).

Concurrently, we detected three immune-related genomic features. SNV neoantigens were measured with NetMHCpan v3.0 ([Nielsen et al., 2016](#_ENREF_16)) by combining OptiType, a highly accurate ensemble HLA genotyping algorithm based on RNA sequencing data ([Szolek et al., 2014](#_ENREF_20)). Fractions of leukocytes were analysed by using a mixture model based on methylation probes with the greatest differences between pure leukocyte cells and normal tissue ([Thorsson et al., 2018](#_ENREF_22)). TCR were explored by the MiTCR ([Bolotin et al., 2013](#_ENREF_1)) and BCR diversity VDJer methods ([Mose et al., 2016](#_ENREF_15)). The information about silent/non-silent mutations was obtained from TCGA. the sub-clonal genome fraction of intratumor mutation was defined by the ABSOLUTE algorithm ([Taylor et al., 2018](#_ENREF_21)).

**Immune landscape**

DDRTree achieved tree graphs based on a combination of multiple parameters which were then used for to depict the general situation of immune subtypes. We differentiate individualized patients along linear trajectories ([Trapnell et al., 2014](#_ENREF_23)), and the complex trajectories with 5 tree structures were analysed by Monocle’s algorithms ([Qiu et al., 2017a](#_ENREF_17); [Qiu et al., 2017b](#_ENREF_18); [Cao et al., 2019](#_ENREF_3)).

**Statistics**

The association between scores of gene module signatures and immune subtypes was examined in the TCGA discovery dataset with Spearman correlation. In the box plots, the bottom and top of the boxes were the 25th and 75th percentiles (interquartile range), the center line corresponded to the median, and the whiskers encompassed 1.5 times the interquartile range. A contingency table was constructed, and the chi-squared test was performed. All statistical analyses were performed using the statistical programming environment R or SPSS software (version 3.4.4). Two-sided *P* values < 0.05 were considered significant.

**Reference**

Bolotin, D.A., Shugay, M., Mamedov, I.Z., Putintseva, E.V., Turchaninova, M.A., Zvyagin, I.V., et al. (2013). MiTCR: software for T-cell receptor sequencing data analysis. *Nat. Methods* 10, 813-814.

Brunet, J.P., Tamayo, P., Golub, T.R., Mesirov, J.P. (2004). Metagenes and molecular pattern discovery using matrix factorization. *Proc. Natl. Acad. Sci. U S A* 101, 4164-4169.

Cao, J., Spielmann, M., Qiu, X., Huang, X., Ibrahim, D.M., Hill, A.J., et al. (2019). The single-cell transcriptional landscape of mammalian organogenesis. *Nature* 566, 496-502.

Fogel, P., Gaston-Mathe, Y., Hawkins, D., Fogel, F., Luta, G., Young, S.S. (2016). Applications of a Novel Clustering Approach Using Non-Negative Matrix Factorization to Environmental Research in Public Health. *Int. J. Environ. Res. Public Health* 13.

Fontes, F., Severo, M., Castro, C., Lourenco, S., Gomes, S., Botelho, F., et al. (2013). Model-based patterns in prostate cancer mortality worldwide. *Br. J. Cancer* 108, 2354-2366.

Gautier, L., Cope, L., Bolstad, B.M., Irizarry, R.A. (2004). affy--analysis of Affymetrix GeneChip data at the probe level. *Bioinformatics* 20, 307-315.

Hochberg, Y., Benjamini, Y. (1990). More powerful procedures for multiple significance testing. *Stat. Med.* 9, 811-818.

Huang da, W., Sherman, B.T., Lempicki, R.A. (2009). Systematic and integrative analysis of large gene lists using DAVID bioinformatics resources. *Nat. Protoc.* 4, 44-57.

Hutchins, L.N., Murphy, S.M., Singh, P., Graber, J.H. (2008). Position-dependent motif characterization using non-negative matrix factorization. *Bioinformatics* 24, 2684-2690.

Kapp, A.V., Tibshirani, R. (2007). Are clusters found in one dataset present in another dataset? *Biostatistics* 8, 9-31.

Li, B., Cui, Y., Nambiar, D.K., Sunwoo, J.B., Li, R. (2019). The Immune Subtypes and Landscape of Squamous Cell Carcinoma. *Clin. Cancer Res.* 25, 3528-3537.

Li, T., Fan, J., Wang, B., Traugh, N., Chen, Q., Liu, J.S., et al. (2017). TIMER: A Web Server for Comprehensive Analysis of Tumor-Infiltrating Immune Cells. *Cancer Res.* 77, e108-e110.

Lovmar, L., Ahlford, A., Jonsson, M., Syvanen, A.C. (2005). Silhouette scores for assessment of SNP genotype clusters. *BMC Genomics* 6, 35.

Marisa, L., de Reynies, A., Duval, A., Selves, J., Gaub, M.P., Vescovo, L., et al. (2013). Gene expression classification of colon cancer into molecular subtypes: characterization, validation, and prognostic value. *PLoS Med.* 10, e1001453.

Mose, L.E., Selitsky, S.R., Bixby, L.M., Marron, D.L., Iglesia, M.D., Serody, J.S., et al. (2016). Assembly-based inference of B-cell receptor repertoires from short read RNA sequencing data with V'DJer. *Bioinformatics* 32, 3729-3734.

Nielsen, M., Andreatta, M. (2016). NetMHCpan-3.0; improved prediction of binding to MHC class I molecules integrating information from multiple receptor and peptide length datasets. *Genome Med.* 8, 33.

Qiu, X., Hill, A., Packer, J., Lin, D., Ma, Y.A., Trapnell, C. (2017a). Single-cell mRNA quantification and differential analysis with Census. *Nat. Methods* 14, 309-315.

Qiu, X., Mao, Q., Tang, Y., Wang, L., Chawla, R., Pliner, H.A., et al. (2017b). Reversed graph embedding resolves complex single-cell trajectories. *Nat. Methods* 14, 979-982.

Seiler, M., Huang, C.C., Szalma, S., Bhanot, G. (2010). ConsensusCluster: a software tool for unsupervised cluster discovery in numerical data. *Omics* 14, 109-113.

Szolek, A., Schubert, B., Mohr, C., Sturm, M., Feldhahn, M., Kohlbacher, O. (2014). OptiType: precision HLA typing from next-generation sequencing data. *Bioinformatics* 30, 3310-3316.

Taylor, A.M., Shih, J., Ha, G., Gao, G.F., Zhang, X., Berger, A.C., et al. (2018). Genomic and Functional Approaches to Understanding Cancer Aneuploidy. *Cancer Cell* 33, 676-689.e673.

Thorsson, V., Gibbs, D.L., Brown, S.D., Wolf, D., Bortone, D.S., Ou Yang, T.H., et al. (2018). The Immune Landscape of Cancer. *Immunity* 48, 812-830.e814.

Trapnell, C., Cacchiarelli, D., Grimsby, J., Pokharel, P., Li, S., Morse, M., et al. (2014). The dynamics and regulators of cell fate decisions are revealed by pseudotemporal ordering of single cells. *Nat. Biotechnol.* 32, 381-386.

Zeng, D., Li, M., Zhou, R., Zhang, J., Sun, H., Shi, M., et al. (2019). Tumor Microenvironment Characterization in Gastric Cancer Identifies Prognostic and Immunotherapeutically Relevant Gene Signatures. *Cancer Immunol. Res.* 7, 737-750.

## Supplementary Figures


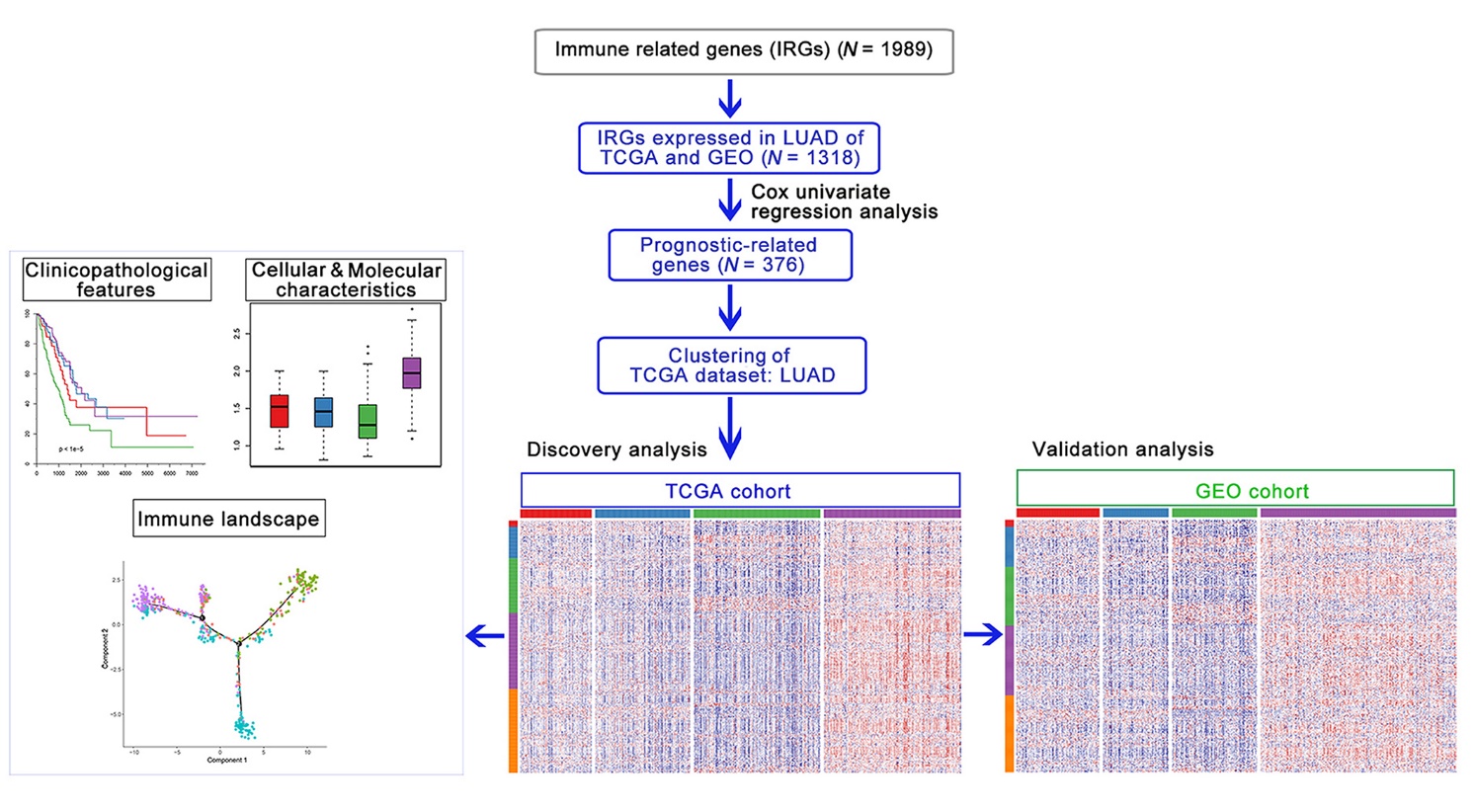


**Supplementary Figure 1.** Workflow of this study. Based on 1989 immune-related genes (IRGs) reported by Li *et al*.([Li et al., 2019](#_ENREF_11)), the genes with no expression and low expression in LUAD were excluded, and 1318 IRGs were screened for study. Next, the 376 genes impacting prognosis of LUAD patients by univariate Cox regression analysis were included in subsequent analysis. Consensus clustering results showed that 4 robust clusters (C1-C4) were identified in the TCGA discovery cohort. We also investigated the reproducibility of the immune subtypes in an independent GEO cohort (GSE68465). Last, we tested the relationship between identified 4 immune subtypes and clinicopathological, cellular and molecular characteristics, and depicted the immune landscape of LUAD.


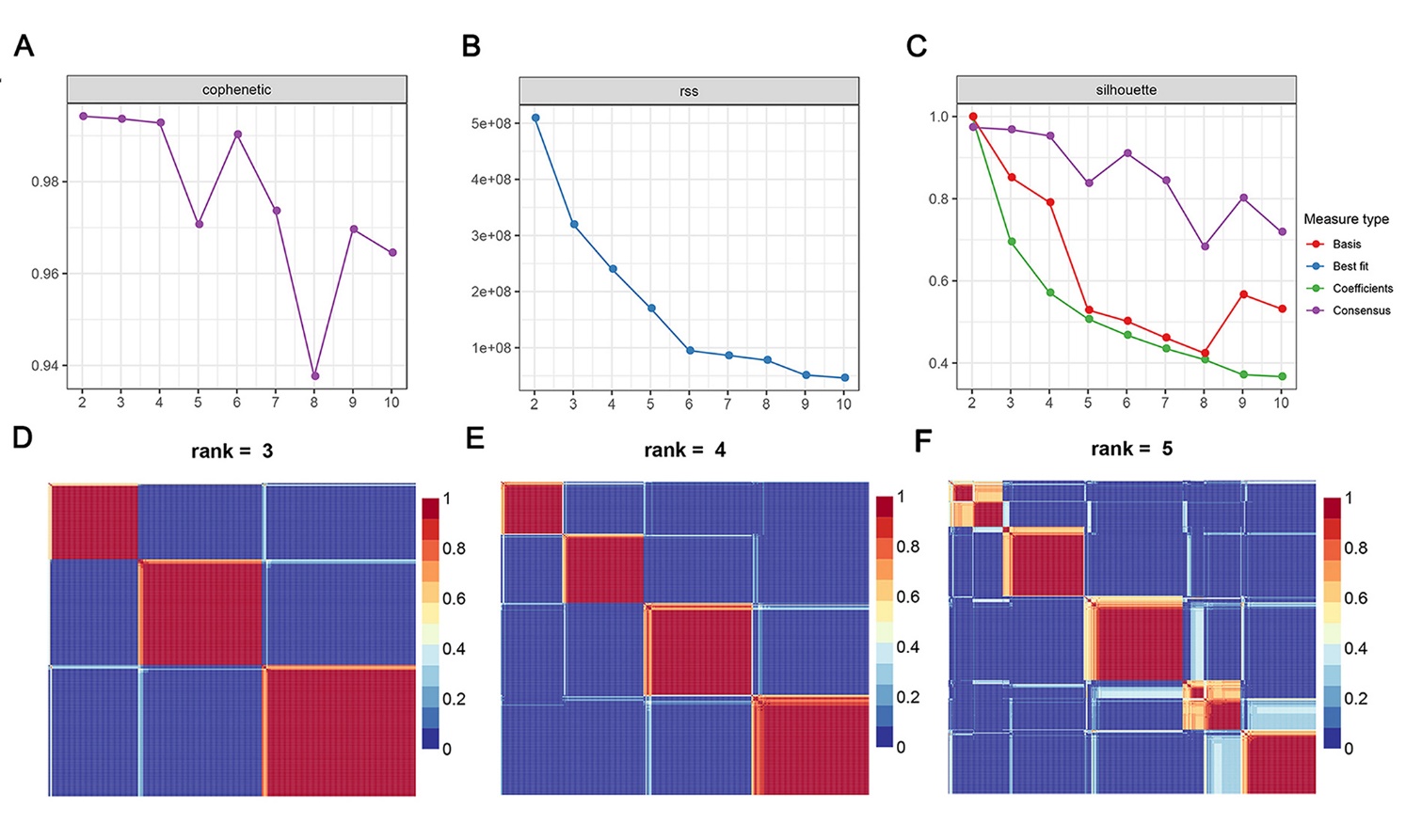


**Supplementary Figure 2.** Nonnegative matrix factorization rank survey of LUAD patients in the TCGA. **(A-C)** Cophenetic, rss and silhouette correlation coefﬁcients for hierarchically clustered matrices computed at ranks 2 to 10. **(D-F)** Correlation matrix heatmaps correspond to ranks 3 to 5.


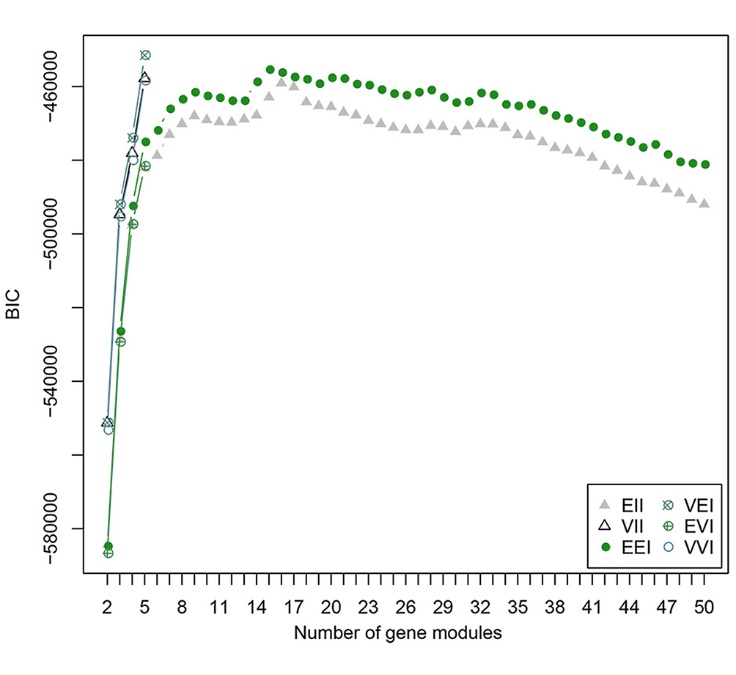


**Supplementary Figure 3.** The Bayesian information criterion (BIC) plot for models fitted to the IRG expression data in TCGA**.** Plot of BIC calculated with different models according to the number of subtypes. EII: spherical with equal volume and equal shape, VII: spherical with variable volume and equal shape, EEI: diagonal with equal shape and equal volume, VE: diagonal with variable shape and equal volume, EVI: diagonal with equal shape and variable volume, VVI: diagonal with variable shape and variable volume.


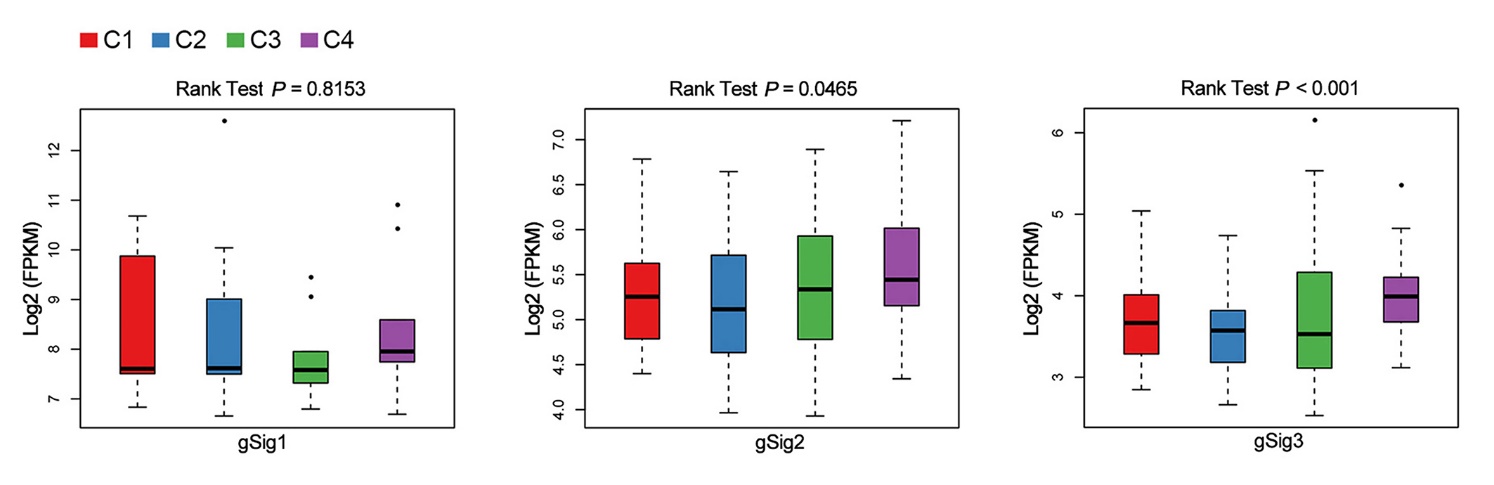


**Supplementary Figure 4.** The correlation between immune subtypes and gSig1, gSig2 and gSig3 in TCGA. Box plots showing expression levels of **(A)** gSig1, **(B)** gSig2, and **(C)** gSig3 in C1-C4.


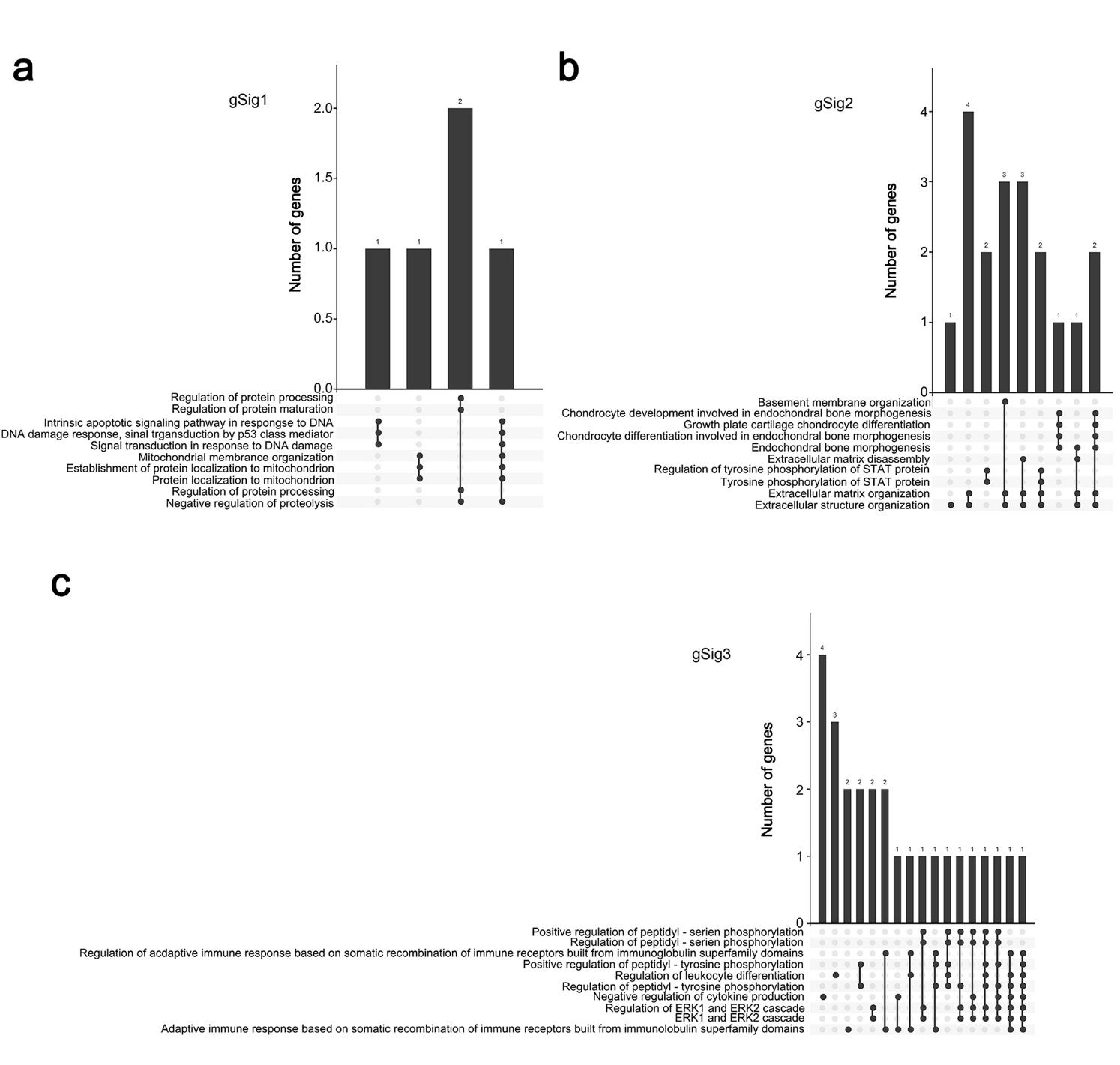


**Supplementary Figure 5.** Gene Ontologies (GO) enrichment of gSig1, gSig2 and gSig3 in TCGA. UpSet plot shows the significant enrichment of Gene Ontologies (GO) of **(A)** gSig1, **(B)** gSig2, and **(C)** gSig3.


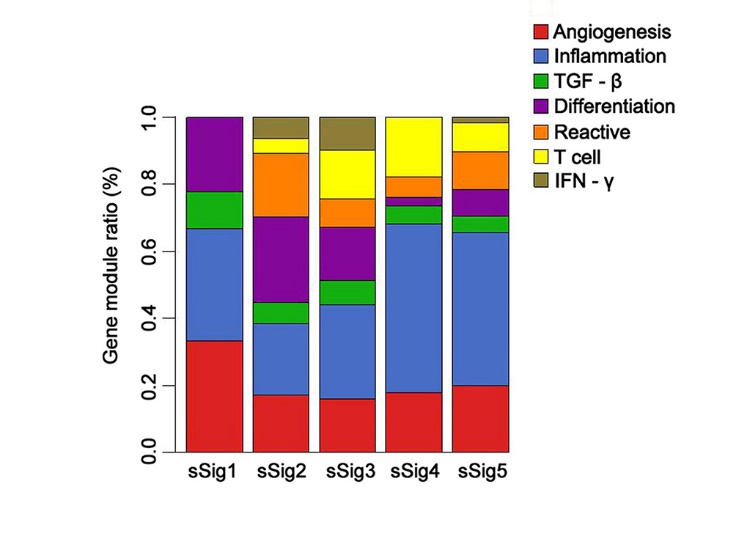


**Supplementary Figure 6.** Characterization of gene module signatures in TCGA. Distribution of 7 gene modules among gene module signatures.


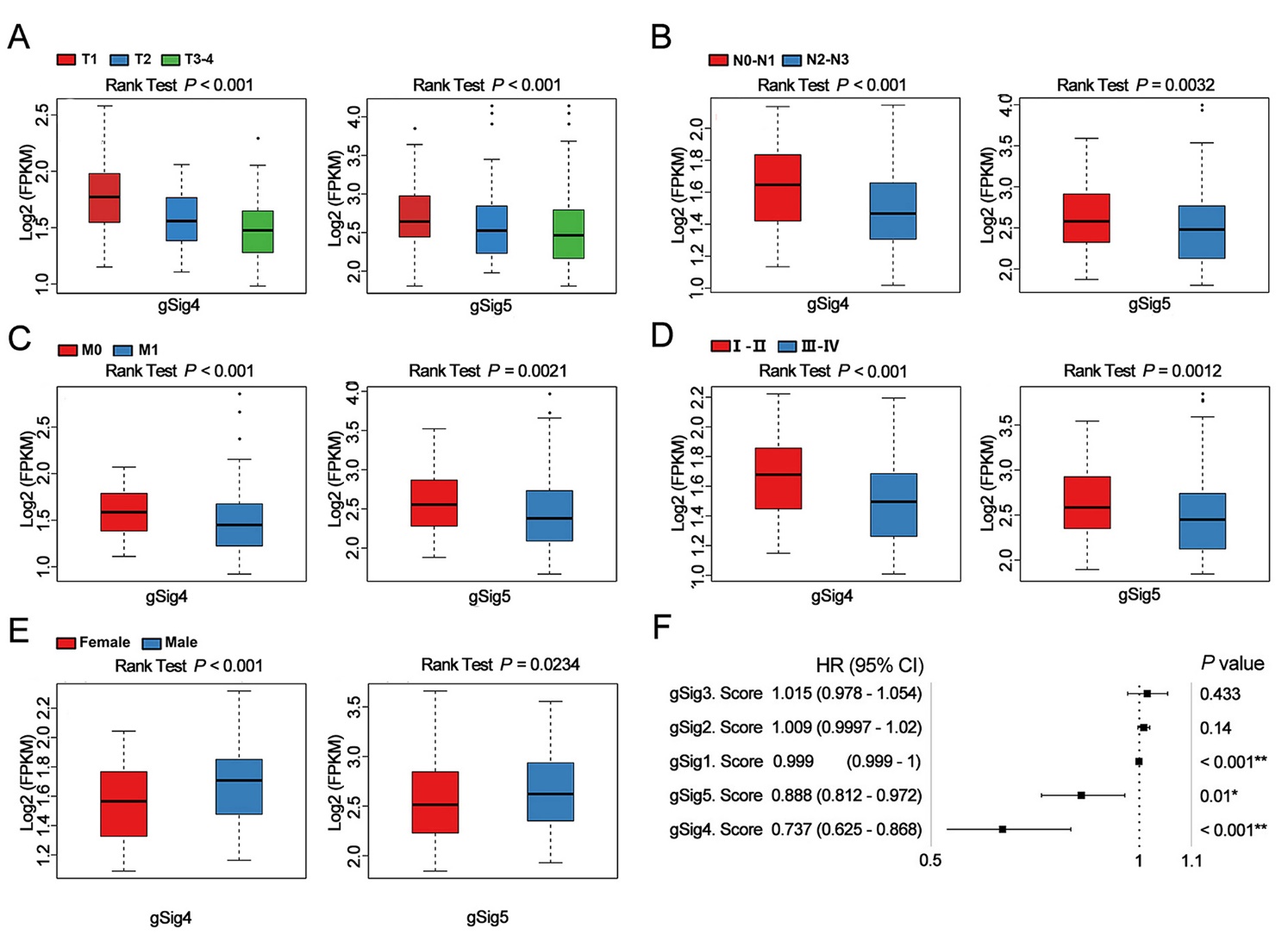


**Supplementary Figure 7.** The relationship between clinical features and gSig4-5 in TCGA. Distribution of **(A)** T stage, **(B)** N stage, **(C)** M stage, **(D)** TNM stage, and **(E)** gender correlated with gSig4 or gSig5. **(F)** Forest plot of hazard ratio by univariate Cox regression for gene signature scores in the TCGA cohort. The size of the box is inversely proportional to the confidence interval. Gene module signatures were treated as continuous variables and *P* values for the Wald test were *p*rovided.


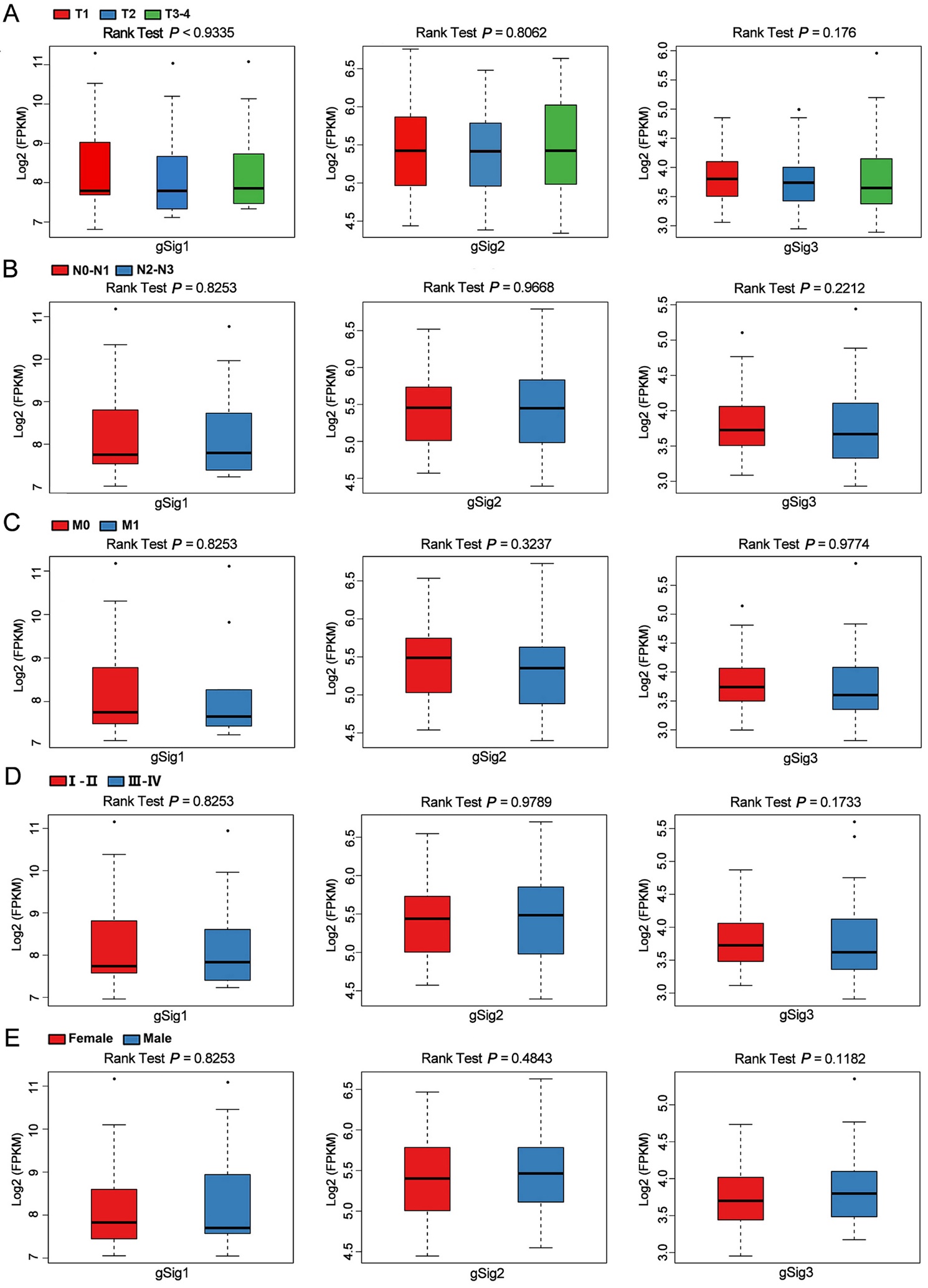


**Supplementary Figure 8.** The relationship between clinical features and gSig1-3 in TCGA. Distribution of **(A)** T stage, **(B)** N stage, **(C)** M stage, **(D)** TNM stage, and **(E)** gender correlated with gSig1-3.


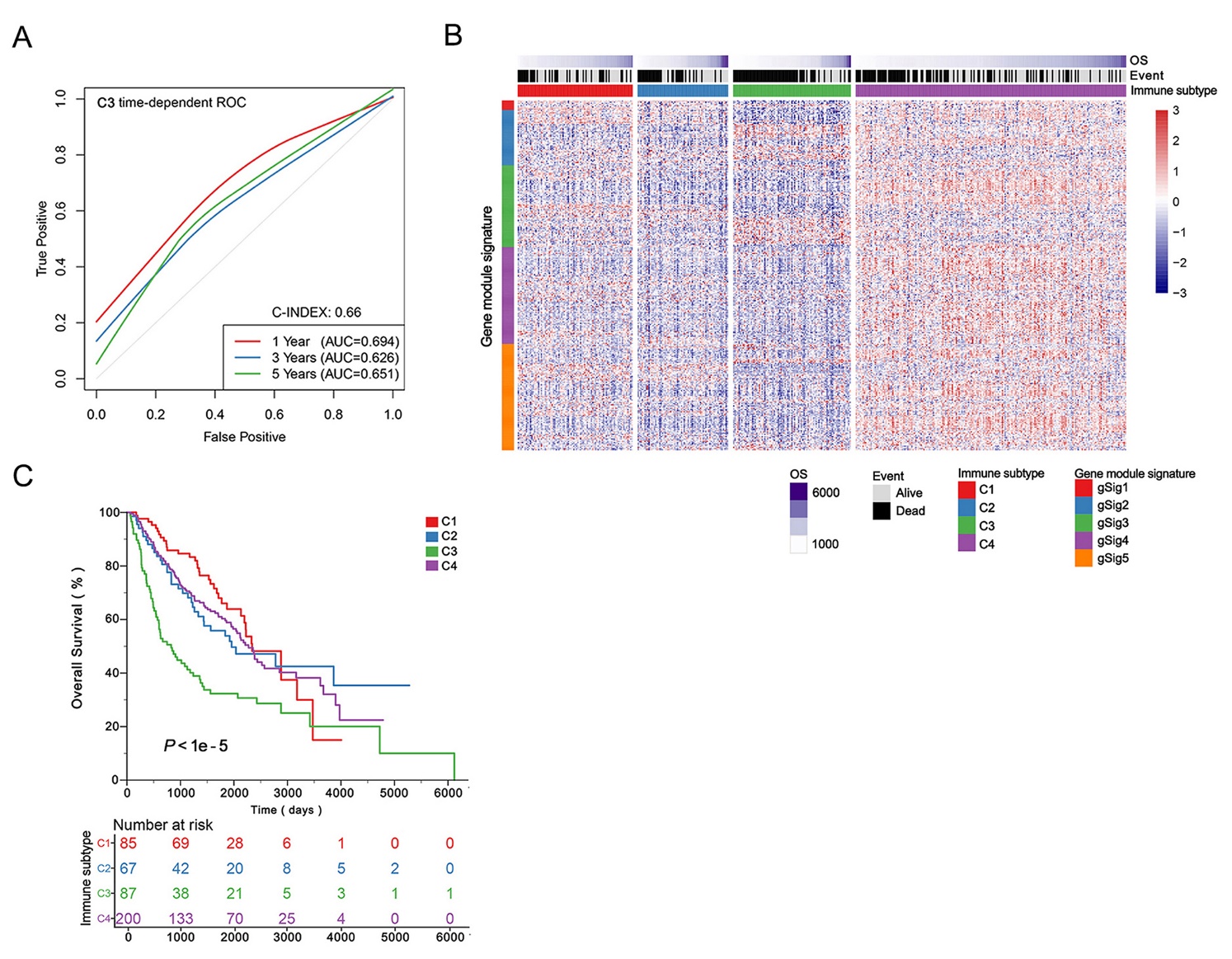


**Supplementary Figure 9.** Receiver operating characteristic curves (ROCs) and verification of immune subtypes. **(A)** ROCs for C3 immune subtype in TCGA. **(B)** The immune subtype and gene module signatures of patients in the validation cohort are depicted in the GEO (GSE68465) cohort. **(C)** Kaplan-Meier curves for OS in the GEO (GSE68465) cohort.

## Supplementary Tables

**Table S1. Clinicopathological characteristics of patients from the TCGA cohort**

**Table S2. Clinicopathological characteristics of patients from the GEO cohort (GSE68465)**

**Table S3. Univariate analysis of immune-related genes in the TCGA cohort**

**Table S4. The immune-related genes in gene module signatures**

**Table S5. The correlation between immune subtypes and gene module signatures**

**Table S6. The GO enrichment analysis of gene module signatures**

**Table S7. The correlation between gene module signatures and gene modules**

**Table S8. The cellular features across immune subtypes in TCGA**

**Table S9. The various molecular features across immune subtypes in TCGA**

**Table S10. The distribution of individual patients in 5 tree structures and 4 identified immune subtypes**
